# Comparison of Single Atoms vs. Sub-Nanoclusters as Co-Catalysts in Perovskites and Metal Oxides for Photocatalytic Technologies

**DOI:** 10.3390/nano15030226

**Published:** 2025-01-30

**Authors:** Anastasia V. Spyrou, Konstantinos Zodhiates, Yiannis Deligiannakis

**Affiliations:** Laboratory of Physical Chemistry of Materials & Environment, Department of Physics, University of Ioannina, 45110 Ioannina, Greece; anast.spirou@yahoo.gr (A.V.S.); zodhiatesk@gmail.com (K.Z.)

**Keywords:** single atom co-catalyst, sub-nanoclusters, quantum-sized small particles, perovskite oxides, photocatalysis

## Abstract

Adatoms as co-catalysts may play a key role in photocatalysis, yet control of their exact configuration remains challenging. Specifically, there is converging evidence that ultra-small structures may be optimal as co-catalysts; however, a comprehensive distinction between single atoms (SAs), sub-nanoclusters (SNCs), and quantum-sized small particles (QSSPs) has yet to be established. Herein, we present a critical review addressing these distinctions, along with challenges related to the controlled synthesis of SAs, SNCs, and QSSPs; their detection methods; and their functional benefits in photocatalysis. Our discussion focuses on perovskite oxides (e.g., such as ABO_3_, where A and B are cations) and metal oxides (M_x_O_y_, where M is a metal) decorated with adatoms, which demonstrate superior photocatalytic performance compared to their unmodified counterparts. Finally, we highlight cases of misinterpretation between SA, SNC, and QSSP configurations emerging from limitations in high-resolution detection techniques and synthesis methods.

## 1. Introduction

The advancement in photocatalytic technologies plays a pivotal role in addressing global energy and environmental challenges, particularly in the domains of sustainable energy conversion and pollutant degradation [1,2,3,4]. Depending on each catalytic process, different types of photocatalysts can be selected, such as metal oxides and perovskites, which exhibit the desired structural and physicochemical properties. However, these systems appear to face challenges that restrict their overall photocatalytic performance, such as limited light absorption, rapid charge recombination, or unfavorable surface properties that might restrict the transfer of electrons to the final acceptor, e.g., H+ in the case of H_2_ production or reduced forms of HCO_3_^−^/CO_2_ toward CO, HCOOH, and CH_4_. In this context, it is well-anticipated that diligent engineering can be applied to the lattice and/or the surface of a semiconductor, including the incorporation of cocatalysts, which can facilitate photoinduced charge separation and improve reaction kinetics [5,6]. The most common co-catalysts are noble metals such as Pt, Pd, Ag, and Au [7]. Alternatively, recent research has provided encouraging results that non-noble metals, i.e., Cu, Ni, Co, or Sn, can be efficient co-catalysts [8,9,10,11,12]. Furthermore, there is mounting evidence that Single atoms dispersed on the surfaces of semiconducting supports can boost their catalytic activity and selectivity [13], i.e., acting as singe-atom (SA) co-catalysts.

### 1.1. Single Atom Catalysts (SACs) and Single Atom Co-Catalysts (SACCs)

The terminology “single atom catalyst-SAC” was first introduced in 2011 by Zhang et al. [14] based on the discovery that anchoring isolated Pt adatoms as “single-atoms” on FeO_x_ significantly improved the system’s catalytic performance in CO oxidation [14]. The use of single atoms in *photo*catalysis was exemplified by Xing et al. [15], who utilized various noble metal single atoms (Pt, Pd, Rh, and Ru) as co-catalysts anchored on a TiO_2_ substrate for photocatalytic H_2_ production. These single metal atoms were stably dispersed on TiO_2_ and demonstrated superior photocatalytic hydrogen evolution performance compared to the corresponding metallic nanoparticles or clusters [15]. Since then, the field of single atom catalysis for photocatalytic applications has gained substantial attention due to its unique ability to maximize atomic efficiency and enhance catalytic activity, selectivity, and stability [13,16]. Single atoms can demonstrate more efficient utilization *per atom* vs. their nanoparticle counterparts. The importance of highly dispersed single atoms of a co-catalyst on a catalyst’s surface lies in the low coordination environment of atoms, and, more interestingly, the tuning of electronic states and efficient charge transfer between the catalyst and co-catalyst, boosted by strong metal–support interaction (SMSI) phenomena [17]. Operationally, it is useful to distinguish the roles of these adatoms as single atom catalysts (SACs) vs. single atom co-catalysts (SACCs) as follows: single atom catalysts refer to cases where an adatom is the catalyst itself. Typically, in such cases, the support plays an auxiliary role. On the other hand, a single atom co-catalysts refer to cases where the support is the catalyst, e.g., such as in the case of photocatalysis, and the adatom plays the role of co-catalyst.

**Figure 1:**
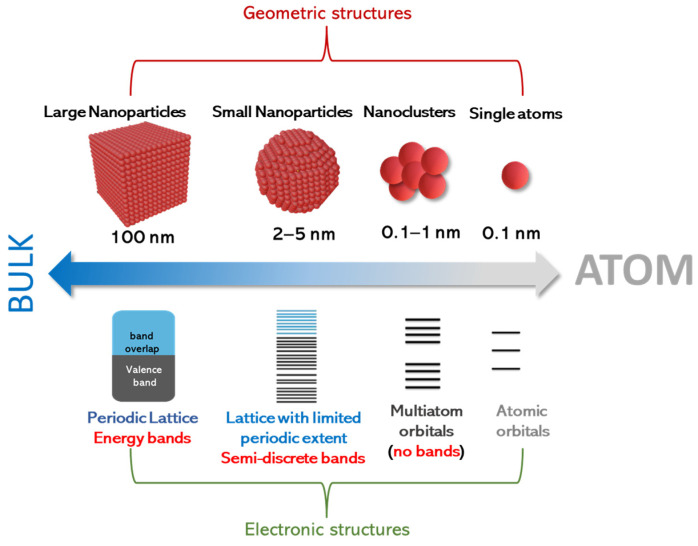
Geometric and electronic structure of single atoms, clusters, small nanoparticles, and larger nanoparticles.

Despite this operational distinction, the physical configuration, and, often, the chemistry and synthesis, of SACs and SACCs may follow similar rules. As shown in Figure 1, in the case of adatoms that are only metal atoms (i.e., not oxides), precise control of their aggregation state is essential for regulating their electronic properties [18,19,20,21]. It is important to notice that, in contrast to previous similar schemes in the literature on the effect of size on electron levels [22,23,24,25,26], in Figure 1, we emphasize the cases of nanoclusters, small nanoparticles, and larger nanoparticles as pertinent configurations that conceptually should be anticipated to be between single atoms and bulk materials. Also, it should be noted that the term “*large nanoparticles*” herein is used to signify that for nanosizes near 100 nm, the electronic properties, e.g., density of states, do not exhibit quantum-sized effects [27]. Thus, we use the term “large nanoparticles” to highlight that, even if these structures are in the “nanoscale”, their properties might tend to resemble those of bulk particles. On a rigorous basis, all these trends can be well documented by theoretical quantum computing tools. However, herein, we present only a conceptual analysis, based on the fundamental concepts of nanoscale solid-state physics [28].

In this context, Figure 1 exemplifies that small nanoclusters do form *multiple energy* levels that do not exist in single atoms. However, in such small nanoclusters, we *do not* have the formation of *energy bands*. This is because small nanoclusters do not favor the stabilization of a periodic lattice; thus, they cannot stabilize Bloch wave functions that would allow for the hybridization of *atomic* orbitals toward *bands* [27]. To obtain a quantitative estimate, geometry-wise, a quantum-sized small particle (QSSPs) of 2 nm that clearly exhibits quantum-size effects would have a number of atoms (N_QSSP_) in the order of ~10^3^ (assuming a typical atomic size of 1–3 Å). This implies that we should consider entities of size < 1 nm as small nanoclusters, with their number of atoms N_SN_ being below N_QSSP_ (i.e., N_SN_ < $10^{2}$ ). This operational distinction helps us classify the energy configuration trends, i.e., the transition from discrete energy multiplicities (not-bands) in small nanoclusters to semi-discrete-bands in quantum-sized small particles to the full-periodic density of states in large nanoparticles with a well-formed periodic lattice.

When these limited-size entities are interfaced with a photocatalyst, interesting phenomena may arise that ultimately allow for the tuning of electronic states of the co-catalyst with the photocatalyst and efficient charge transfer between the catalyst and co-catalyst caused by strong metal–support interaction (SMSI) [29,30,31,32].

**Figure 2:**
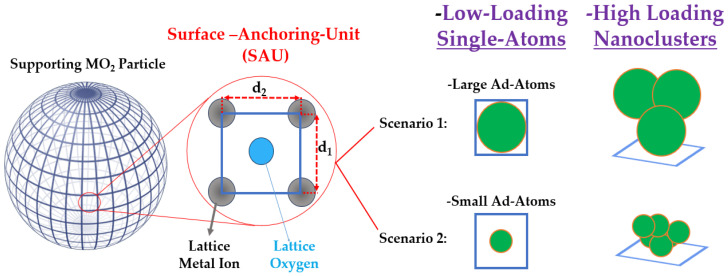
Mode of ad atom anchoring per surface anchoring unit (SAU) on the support. Assuming that anchoring of ad atoms occurs on the O atom for each SAU, then at low loadings, 1 ad atom or less is anchored per SAU. This corresponds to single atom co-catalyst configuration. At higher loadings > 1 ad atom/per SAU will result in nanocluster formation, where the agglomeration degree is influenced by the SAU and the interactions between eh ad atoms. When the interactions between the ad atoms predominantly stabilize larger agglomerates, then small nanoparticles start to be favored.

Determining the optimal loading on a supporting particle is inherently challenging, partly because there can be a significant difference between the *nominal* amount of ad atoms, i.e., used in the precursors during the anchoring process, and the *actual* amount that remains anchored. As shown schematically in Figure 2, the geometrical characteristics of the semiconductor’s surface and the size of the ad atoms allow us to anticipate some limits of single atoms versus nanocluster deposition.

To illustrate this, Figure 2 depicts an ideal spherical particle whose surface is divided into “surface-anchoring-unit” (SAU). In a typical metal oxide, ad atoms preferentially anchor to the oxygen (O) atoms of the lattice. On a simple MO_2_ oxide surface, four metal atoms (M) frame each O atom, defining a SAU that spans an elementary surface of 0.1 to 1 nm^2^, i.e., based on typical atomic diameters of 1–3 Å and M–O bond lengths of 1–2 Å. In this configuration, if one SAU binds one ad atom, that constitutes the single atom scenario; if the number of ad atoms per SAU exceeds one, clusters will inevitably form. In this context, the size of the ad atom itself (see Table 1) will dictate how many atoms can be accommodated at a minimum in a small nanocluster per SAU.

**Table 1:** Parameters of atoms commonly used as ad atoms in photocatalysis.

Ad Atom	Co	Ni	Cu	Sn	Bi	Pd	Ag	Pt	Au
**Most common Oxidation State while on support**	2+	2+	2+	2+	3+	0	0	0	0
Atomic Diameter (Å) *	1.09	1.10	1.14	1.44	1.92	4.04	3.44	1.2	2.74
Atomic Weight **	58.93	58.69	63.55	195.08	106.42	107.87	118.71	196.97	208.98

* Data taken from [33]; ** data taken from [34].

So far, although great progress has been achieved, the precise control of ad atom deposition in photocatalytic systems still faces many challenges, and diligent optimization is required in order to reach their maximum performance. A maximized co-catalyst loading may be targeted (increased surface density) while avoiding single atom agglomeration into sub-nanoclusters or even small nanoparticles [35,36,37]. Interestingly, recent works [38,39] in photocatalytic hydrogen production show that a low ad atom loading can be sufficient for enhanced reaction rate in cases where the semiconductors possess efficient electronic properties. For example, Qin et al. [39] studied the effect of Pt co-catalysts on TiO_2_ semiconductors using different loadings of Pt leading from low to high SACC density. Their data revealed that the optimal Pt loading, i.e., for maximum H_2_ production, is 2 × 10^5^ Pt atoms per μm^2^ of TiO_2_ surface. A further increase in SACC density did not enhance the reaction rate [39]. Schmuki and Wu, in their review [40], clearly emphasized this aspect, discussing that semiconductors with well-defined structures and favorable electronic properties, e.g., materials with high carrier transport and surface harvesting lengths, can significantly reduce the need for a high loading density of Pt atoms. Conversely, using highly defective semiconductors or semi-insulating materials necessitates a greater co-catalyst loading and/or results in underperforming systems [40].

### 1.2. Perovskites in Photocatalysis

Originally, the term “perovskite” was used to describe crystal structures that follow the chemical formula ABX_3_, where A and B are usually metal cations (with A being larger than B), and X is a non-metal anion [41,42]. The structure formed is a cubic unit cell with the A cation occupying the corners, the X anion occupying the face-centered positions, and the B cation occupying the middle of the cube [42]. This configuration is highly symmetric, with different patterns of periodicity depending on the lattice point of reference. Over the years, the group of perovskites has been expanded to fit all compounds that form the perovskite structure without strictly obeying the ABX_3_ chemical formula [43]. The wide category of ABX_3_ used in photocatalysis includes perovskite-oxides like ABO_3_. This gave rise to new perovskite genres like high-entropy perovskites, e.g., La(Mn_0.2_Co_0.2_Fe_0.2_Ni_0.2_Cr_0.2_)O_3_ [44] and A-site deficient perovskites, e.g., La_0.6_Sr_0.2_Ti_0.85_Ni_0.15_O_3−−δ_ [45]. In addition, non-oxide perovskites have been developed, e.g., halide perovskites. CH(NH_2_)_2_PbBr_3−x_I_x_ [46,47].

Perovskite-oxides, ABO_3_, are usually semiconductors [48]. Strontium titanate (SrTiO_3_), for example [26,49], a widely used perovskite-oxide photocatalyst [50], forms a valence band predominantly comprised of oxygen 2p orbitals hybridized with states from the A-site (Sr) and B-site (Ti) cations, while the conduction band is mainly composed of the transition metal B-site cation’s 3d t_2g_ and e_g_ orbitals [51]. Titanium is in a Ti^4+^ oxidation state (3d^0^ configuration), which results in a fully occupied valence band and a completely empty conduction band [52]. Consequently, the Fermi level sits at the top of the valence band. However, the energy band structure discussed above concerns an ideal perovskite structure without defects. If defects were to be considered, they would add mid-gap states, which would allow some electrons to jump to the conduction band [53]. Therefore, the Fermi level would shift toward the middle of the band gap, charge separation would become more efficient, and the semi-conductor properties of the material would further improve [54,55,56]. In general, most perovskite-oxides, like ABO_3_, exhibit an electronic structure where the valence band is predominantly formed by oxygen 2p orbitals and the conduction band mainly consists of transition metal d orbitals [57]. As a result, the band gap is highly tunable through compositional variations and defect engineering, making perovskites favorable for catalytic applications [58].

**Figure 3:**
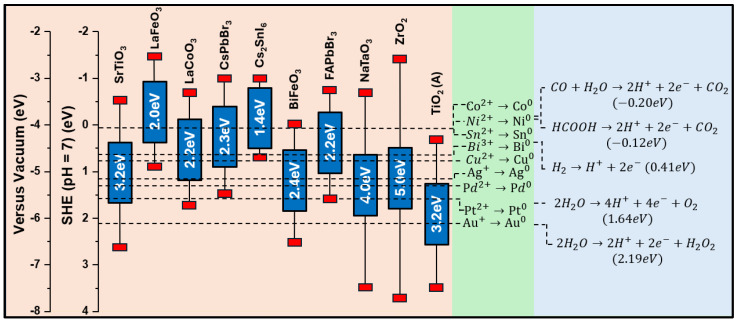
Band structure of several perovskite photocatalysts, including ZrO_2_ and TiO_2_ as reference single metal oxides, are displayed on the right side of the figure. The redox potentials of selected atoms, commonly used as ad atoms/co-catalysts, are also shown in the middle of the figure. Some fundamental catalytic reactions, followed by photocatalysis, are displayed on the right side of figure. Redox potential values were obtained from [59,60,61,62,63,64,65,66]. Notice that all redox values in (eV) in the Y-axis are [T = 25 °C] vs. the standard hydrogen electrode at pH = 7, i.e., all Y values are offset by +0.59 mV × pH vs. the value at pH = 0.

As a result, perovskites may possess favorable light-absorption properties [67], high extinction coefficients, and extended charge-carrier diffusion lengths, allowing them to efficiently capture light and transport photoinduced charge carriers over relatively long distances to surface sites for redox reactions [67]. Furthermore, in this context, perovskites, characterized by their high thermal stability and optimal crystal structure, have been shown to significantly improve the stability of precious metals, making them excellent candidates as supports for developing single atom catalysts [68,69].

*Aspects of Energetics of Photocatalysts and Single Atom Co-catalysts*: Photocatalytic reactions can be considered to consist of reduction and oxidation. In this context, it is important to view the electronic properties of perovskites as well as their decorating ad atoms. In Figure 3, we summarize the energy states (at pH = 7) of some prominent perovskite and oxide materials, along with the redox energies of several single atoms that are commonly used as ad atoms/co-catalysts in conjunction with the metal oxide perovskites. In addition, in Figure 3, some fundamental redox reactions (pH = 7), typically created via photocatalysis, are presented.

Some combinations of these materials decorated with single atoms have been studied to develop a comprehension of the underlying energetics. Two Y-axes are present in the graph: one represents the redox energy scale versus the standard hydrogen electrode (SHE) at pH = 7, and the other is versus the vacuum energy scale. Both scales serve a different analysis purpose but are of equal importance. The redox energy scale was created as a reference for all catalytic procedures that occur in aquatic environments. This scale offers the flexibility of altering the pH of the aquatic solution, i.e., the Nernst equation [70] implies that for every unit increase in pH, one must add 59 mV per electron to the potentials at scale. In Figure 3, a SHE pH = 7 scale was used, and the 7 points of increased pH caused a +0.413 eV shift to all values. Thus, the hydrogen oxidation reaction is +0.41 eV at pH = 7. While reading values of the redox energy scale, one must bear in mind that the more positive an energy level is, the more oxidizing the environment it creates, while the more negative a redox energy level gets, the more reducing its environment becomes. The vacuum scale is referenced to the commonly accepted value of energy required to excite an electron to the point it goes far away from any interaction (to the vacuum). This energy level is, by definition, located at 0 eV on the vacuum scale, and it corresponds to −4.44 eV in the redox energy scale vs. SHE at pH = 0 scale, i.e., −4.03 eV at pH = 7.

Regarding the positioning of the ad atoms/co-catalysts in Figure 3, more positive redox energy values indicate atoms requiring a strong oxidizing potential to be stabilized in their ionic form. Otherwise stated, atoms located at the bottom of the redox energy scale are more easily stabilized than their M^0^ metal state. Thus, atoms of Ag and Pt on oxide surfaces tend to be stabilized in their reduced M^0^ metal state. This is determined by the local redox potential, which is created locally at the interface of the ad atom and the metal oxide photocatalyst. Although Pd and Ag are noble metals, they are more prone to oxidation than Au or Pt. This has been elucidated recently for the case of Pd on TiO_2_, where we discovered multiple Pd oxidation states [71], i.e., Pd^3+^, Pd^2+^, and Pd^1+^, formed depending on the layer thickness of Pd on TiO_2_ [71]. Using electron paramagnetic resonance as high-resolution spectroscopy, we demonstrated that ultrafine, single atom Pd deposition formed Pd^0^/Pd^1+^, while thicker nanoclusters also contained Pd^2+^/Pd^3+^ states [71]. These Pd-ad atoms have been shown to be highly active in H_2_ photocatalysis and catalytic H_2_ production from HCOOH [72]. On the other end, atoms with redox energy at more negative values in Figure 3 require very negative potentials to be reduced, and thus they tend to remain in their oxidized states. Consequently, when deposited on the photocatalyst surface, they will, in principle, be found in their cationic state.

These trends can be altered by the redox action of the photoinduced holes and electrons, which possess the redox energies of the corresponding VB-top and CB-bottom of their parent semiconductor (see Figure 3). Thus, semiconductors with highly reducing CB (e.g., NaTaO_3_, SrTiO_3_, etc.) should generate electrons in their CB that are capable of reducing Ni^2+^ to Ni^0^, Co^2+^ to Co^0^, and Cu^2+^ to Cu^0^. On the other hand, TiO_2_ with less-reducing CB cannot reduce Ni^2+^ to Ni^0^ or Co^2+^ to Co^0^, but it can reduce Cu^2+^ to Cu^0^. Meanwhile, all the semiconductors in Figure 3 should be able to donate electrons to Au, Pt, Pd, and Ag.

In the same context, the reduction of CO_2_ to HCOOH is only favorable for the high-CB semiconductors and less so for TiO_2_ and BiFeO_3_. It is important to take into account that interfacial energetics can change these considerations drastically, i.e., the energy redox states of CO_2_/HCO_3_/HCOOH may differ at the interface with a semiconductor, and therefore, the efficiency of CO_2_/HCO_3_ photocatalytic reduction toward HCOOH can be significantly influenced.

As mentioned above, perovskites possess flexible structural and electronic properties divergent from their parental metal oxide, which makes them desirable for various photocatalytic applications. For example, as shown in the redox energy diagram (Figure 3), the TiO_2_ (Anatase) semiconductor possesses a mildly reducing CB edge, with a band gap of 3.2 eV. After perovskitization, in which TiO_2_ transforms into ABO_3_-type SrTiO_3_, the conduction band is shifted toward more negative redox energies. However, due to their special chemistry, perovskite synthesis is quite demanding, as it requires the simultaneous incorporation of two cations, A and B, into the ABO_3_ lattice with the precise stoichiometric ratio of 1:1:3 [73,74]. Consequently, the control of atomic dispersion of single atoms or sub-nanoclusters on their surface is more complex compared to metal oxide substrates.

**Figure 4:**
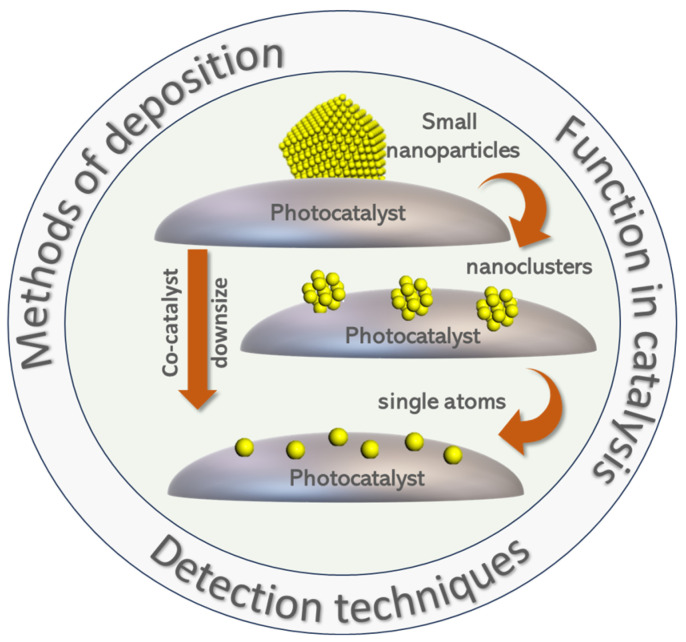
The main challenges concerning SAs/NCs/QSSPs as co-(photo)catalysts: methods of deposition on the substrate, characterization techniques used for the detection of the aggregation state of the co-catalyst, and their function in various photocatalytic applications.

In the present review, we discuss the main key challenges in achieving efficient interfacing of a photocatalyst with single atoms, sub-nanoclusters, or quantum-sized small particles. We discuss three different aspects (as shown in Figure 4):

1. *The synthesis methods*. Due to the demanding nature of the controlled formation of SAs vs. NCs vs. QSSPs and their anchoring on the catalytic particle, synthesis poses standalone challenges. This may result in a mixture of SAs and NCs or NCs and QSSPs. We discuss cases where these issues are not clear or there are misassignments of SAs to NCs or NCs to QSSP and vice versa.
2. *The detection–analysis methods*. The *de facto* low-atom concentration required to form SAs, NCs, or QSSPs excludes their detection by traditional material characterization methods, e.g., X-ray diffraction, and requires specific spectroscopic microscopy techniques, often a combination of them. We discuss examples where a specific method provides the most convincing data on the distinction between SAs, NCs, and QSSPs.
3. *The performance characteristics*. The gain in performance determines whether SA, NCs, or QSSPs are preferable for a specific photocatalytic system. We discuss examples where a clear benefit is achieved by a certain configuration and discuss possible trends and generalizations, if allowed by the data.

For coherence, this review focuses on metal atom co-catalysts interfaced with metal oxides as photocatalysts. Specifically, we focus on metal-oxide perovskite photocatalysts, e.g., of the type ABO_3_, since they show high-premise and higher photocatalytic efficiency vs. the corresponding single-metal oxides [48]. For completeness, we include selected non-oxide perovskites as pertinent examples to discuss the synthesis or the detection method for the co-catalyst and their role.

## 2. Synthesis Methods

The fundamental concept of a single atom co-catalyst depends on achieving this highly dispersed state of isolated single metal atoms anchored on suitable supports. This mechanism is not trivial, as metal atoms possess high surface free energy and therefore tend to aggregate into clusters or even nanoparticles during synthesis or subsequent treatments. In order to navigate toward successful single atom anchoring, synthetic strategies must adopt strong metal–support interactions and often involve carefully selected modifications of the support. For instance, lattice or surface defects in TiO_2_ and vacant sites have been reported as prominent options to stabilize single Pt atoms on the support’s surface [75,76,77,78]. Defects may prevent atom aggregation via the promotion of the formation of strong bonds between single atoms [79]. For instance, a DFT study on the surface of Fe_3_O_4_ demonstrated that oxygen vacancies favor the anchoring of single, noble metal atoms [76]. Awide range of synthetic approaches have been employed to prepare SACCs, encompassing both wet and dry chemistry categories, each with its own advantages and drawbacks that influence factors such as uniform distribution and overall catalyst stability. Metal loading is another critical parameter influenced by the synthesis method. Currently, most practices resort to low metal loading (0–2 wt.%), to minimize aggregation. Thus, further advancements in synthesis techniques and support engineering will be necessary. Alternatively, flame spray pyrolysis [73] is an industrial-scale approach to achieve precise control over the size and structure of the catalyst and co-catalyst. Furthermore, due to the high temperatures involved in the synthesis, strong metal–support interactions occur between the co-catalyst and the support [80].

### 2.1. Wet Chemical Synthesis Methods

Wet chemical synthesis methods for single atom co-catalysts (SACCs) typically involve dispersing metal precursors within a liquid medium and subsequently anchoring individual metal atoms onto a support under carefully controlled conditions [31]. Such approaches (see Table 2), which include wetness impregnation, adsorption, and strong electrostatic adsorption, can produce highly dispersed single atoms by promoting strong metal–support interactions and employing tailored reaction environments [31,75]. However, since metal atoms inherently tend to aggregate into clusters or nanoparticles, controlling reaction kinetics is critical. Key parameters such as pH, precursor concentration, and reaction temperature must be closely monitored [76]. Various techniques, including incipient wetness impregnation and strong electrostatic adsorption, can enhance spatial confinement and maintain high dispersion [75]. Although these methods often produce efficient catalysts, long-term stability may still pose a challenge [75]. To address this, post-treatment methods such as controlled reduction and pyrolysis have been employed [81].

### 2.2. Dry Chemistry Synthesis Methods

Dry chemistry methods are prominent strategies [31] for synthesizing single atom catalysts (SACCs) by achieving confined atomic dispersion on support. The dry chemistry mechanisms typically rely on controlled physical and chemical processes to anchor single metal atoms without involving solution-phase reactions. The most widespread examples of these methods are Atomic Layer Deposition (ALD) and pyrolysis [82,83,84,85]. ALD is characterized by the sequential exposure of support to metal precursors, enabling the precise deposition of single atoms through controlled ligand exchange reactions and surface binding [84]. This method provides high uniformity, atomic precision, and tunability of active site density, making it a valuable approach for producing stable SACCs [83]. On the other hand, pyrolysis methods [81] involve the thermal decomposition of metal–organic precursors under controlled atmospheres. Dry chemistry methods are notable for their ability to generate high-purity SACCs with exceptional structural stability, though challenges such as scalability and high energy demands remain to be addressed.

**Table 2:** Literature summary of the synthesis methods used for single atom deposition on various substrates.

Nanostructures/ Substrates	Co-Catalyst	Synthesis Method	Classification of Synthesis Method	Ref.
LaFeO_3_	Au	Deposition Precipitation	Wet chemistry	[68]
CsPbBr_3_	Pt	Photo-deposition	Wet chemistry	[86]
SrTiO_3_	Pt	Wet Impregnation	Wet chemistry	[87]
Cs_2_SnI_6_	Pt	Wet Impregnation	Wet chemistry	[88]
CsPbBr_3_	Pt	Photoreduction	Wet chemistry	[89]
FAPbBr_3−x_I_x_ (FA = CH(NH_2_)_2_)	Pt	Co-precipitation	Wet chemistry	[90]
NaTaO_3_	Ni	Flame Spray Pyrolysis	Dry chemistry	[74]
LaCoO_3_	Pt	Solution-Mediated Adsorption	Wet chemistry	[91]
SrTiO_3_	Ni	E-Beam Evaporation	Dry chemistry	[92]
BiFeO_3_	Co	Immersion	Wet chemistry	[93]
SrTiO_3_	Pd	Photo-deposition	Wet chemistry	[94]
SrTiO_3_	Au	Sputtering	Dry chemistry	[95]
TiO_2_	Cu	MOF synthesis	Wet chemistry	[96]
TiO_2_	Cu	Wet Impregnation	Wet chemistry	[97]
TiO_2_	Pd	Flame Spray Pyrolysis	Dry chemistry	[71]
ZrO_2_	Ni	Solvothermal	Wet chemistry	[98]
ZrO_2_	Pt	Wet-Impregnation	Wet chemistry	[99]

## 3. Characterization Techniques for Detection of SACCs/NCs/QSSPs

**Figure 5:**
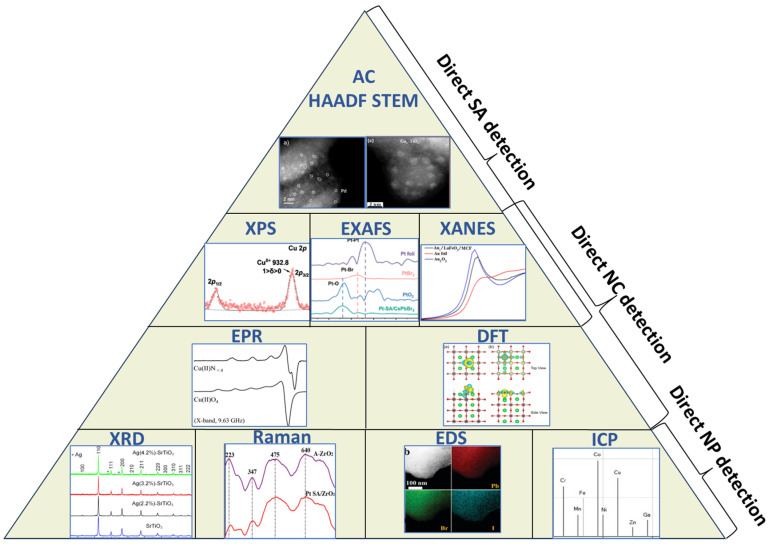
Characterization techniques used for the detection of single atoms, nanoclusters, and small nanoparticles. AC HAADF-STEM: reproduced with permission from ref. [94]. Copyright {2024} Wiley-VCH. XPS: Reprinted (adapted) with permission from [97]. Copyright {2024}, American Chemical Society. EXAFS: Reprinted from [89] with permission from Elsevier. XANES: Reprinted from [68] with permission from Elsevier. EPR: Reprinted from [108] with permission from Elsevier. DFT: Reproduced from [109] with permission from the Royal Society of Chemistry. XRD: Reproduced from Ref. [110] with permission from the Royal Society of Chemistry. Raman: Reprinted from [99] with permission from Elsevier. EDS: Reproduced from Ref. [90] with permission from the Royal Society of Chemistry. ICP: Reproduced with permission from ref. [111]. Copyright {2016} Wiley-VCH.

One of the most challenging aspects of creating SACCs or NCs is to prove the atom anchoring on the support’s surface [100,101,102]. This objective pushes current characterization techniques and analyzation methods to their limits and it usually requires a combination of them in order to produce the desirable results [103] (see Figure 5). However, even with many techniques and methods in hand, it might still be difficult to determine the outcome of the single atom anchoring [102]. This is because not all single atoms have the same fate once they get on the surface of the support [75]. Some atoms might develop a strong metal–support interaction and remain as single atoms [104], but others might aggregate into clusters or nanoparticles or even infiltrate the support’s lattice [94]. These are all possible outcomes, and statistically speaking, they can all occur at once [103]. Nevertheless, each case becomes observable only when it accumulates a major percentage over the others. Bearing in mind that, in most studies, the SACCs’ loading percentage usually does not exceed 2.0 wt.%, one must be very aware of local phenomena while examining an SACC sample.

### 3.1. Indirect Characterization of SACCs/NCs

The characterization techniques used for SACCs/NCs can be divided into two major categories (see Figure 5): techniques that provide direct and indirect proof of single atoms [102,103]. The first group is comprised of procedures that can interact directly with single atoms [95,105] and provide data accordingly, and the second group can only exclude undesired outcomes [101]. For instance, aberration-corrected high-angle annular dark field scanning transmission electron microscopy (AC HAADF-STEM) can picture single atoms on the support’s surface if the Z contrast is favorable [95], but an X-ray diffraction (XRD) pattern can only exclude the formation of nanoparticles. This information is not very helpful as it only excludes one possible outcome, but it is necessary, nevertheless. Another technique that provides indirect data is Energy Dispersive Spectrometry (EDS) [96,99,103]. Although detecting the presence of single atoms is beyond its limits, EDS can either confirm or exclude the formation of clusters (and nanoparticles) by detecting areas with high aggregation (hot spots). In addition, it can provide information on the uniform and dispersed atom distribution on the support’s surface, which is favorable for single atom anchoring. However, EDS provides localized data, and it can be misleading. To overcome this uncertainty, another technique could be utilized that gives information on the total loading on the support. For instance, Inductively Coupled Plasma (ICP) spectrometry can provide the total loading percentage (per weight) of the desired element [96,106]. This percentage is usually around 2 wt.%, but it can vary depending on the supporting material. If a system is known to support up to 1.5 wt.% of single metal atoms and the ICP gives a loading percentage of 5 wt.%, it should raise suspicions about cluster formation. Lastly, Raman Spectroscopy can be utilized to study the vibrational modes of the support material [107]. Shifts in peak positions, intensities, and line shapes can be interpreted as local structure changes caused by the bonding of atoms on the surface. The shifts might be subtle, but if systematic in the Raman spectrum, they can provide supporting evidence [99].

### 3.2. Direct Characterization of SACCs

*AC HAADF-STEM*: Aberration-corrected high-angle annular dark-field scanning transmission electron microscopy (AC HAADF-STEM) operates by focusing a finely tuned, aberration-corrected electron probe onto a thin, electron-transparent sample and collecting scattered electrons at high angles using an annular dark-field detector [68,96]. The electron scattering in the sample is proportional to the square of the atomic number (Z) of the scattering atom [103]. Heavier elements scatter electrons more and therefore appear brighter in AC HAADF-STEM imaging than lighter elements (see Figure 5). For ideal imaging, heavy elements (such as Pt and Au) should be anchored on a support comprising lighter elements. The bigger the difference in the atomic number, the greater the contrast. For instance, a study by J. Jones et al. reported Pt deposition over TiO_2_ or CeO_2_. Pt has an atomic number of 78, Ti 22, O 8, and Ce 58. While imaging Pt single atoms on TiO_2_ anatase, Pt appears 13 times brighter than the Ti atom (corresponding to the thickness variation of 3–4 nm along the (001) orientation) [112]. When the substrate was changed to CeO_2_, the contrast was way lower (corresponding to the thickness variation of ~0.5nm in the support) [113]. Consequently, AC HAADF-STEM in [112] could directly detect single atoms if the contrast with its support is adequate. An additional observation was that the sample examined must be sufficiently thin (e.g., <50 nm), and the area of interest must be at the edge of the support in order to have sufficient electron scattering [112].

Other complexities arise from surface morphology and thickness variations, as these can produce local bright signals unrelated to metal species [100]. To address such issues, the relative contrast between the metal atoms and the support must be carefully evaluated, sometimes through image simulations, complementary spectroscopic analysis (e.g., EDS or EELS), and comparison with known reference samples [103]. As such, AC HAADF-STEM, when combined with careful data interpretation, can provide compelling evidence for the presence of isolated metal atoms and their dispersion states on various supports, thereby aiding in the identification and characterization of SACCs.

SEM is also pertinent in SA research. Although SEM cannot directly visualize single atoms, given that the resolution is generally insufficient for sub-nanometer features, it can be employed to confirm the absence of nanoparticles above its resolution limit (~ 1 nm in modern Field Emission-SEM instruments). Thus, if another technique (e.g., XPS) confirms Pt is present in the sample, but Pt particles are not visible by SEM, this indirectly supports the presence of sub-nanoclusters or single atoms. Furthermore, the combination of FE-SEM and XPS can efficiently scan large surfaces to compare nanoparticle and single atom contributions, facilitating rapid parameter screening during catalyst development [40].

*X-ray Absorption Spectroscopy (XAS)*: X-ray Absorption Spectroscopy is an umbrella term for techniques that probe the local electronic and structural environment of an element within a material [101]. The main mechanism begins with the tunable X-ray radiation of the sample. As the energy of the X-rays is scanned across an absorption edge of interest, a portion of them will be absorbed and promote core electrons to unoccupied states (the near-edge region) or into the continuum (extended region) [114]. The first depends much on the density of unoccupied states and can therefore reveal changes in the oxidation state of the atom. The latter results in the emission of photoelectrons that will be scattered by neighboring atoms and interfere with each other either constructively or destructively [114]. This will create a fingerprint of the local coordination environment. Consequently, the absorption spectrum can be broken down into X-ray Absorption Near Edge Structure (XANES) and Extended X-ray Absorption Fine Structure (EXAFS) [114].

XANES examines the absorption spectrum near the absorption edge of an element, providing insights into the oxidation state, electronic configuration, and coordination environment of the absorbing atom [114]. Single atom catalysts and small nanoclusters often display unique near-edge features, such as shifts in the edge energy or changes in the intensity of the “white line”, that differ from clusters and bulk materials [100]. The shift occurs because isolated atoms have distinctive arrangements for the valence electrons and better interaction with the support [102]. For instance, the “white line” intensity can reflect the number of unoccupied d-states, which differs when a metal atom gets anchored as an isolated atom because of the altered oxidation states [94]. By comparing the spectral fingerprints with well-studied references, one can find evidence of distinct electronic environments associated with single atom dispersion [86].

EXAFS analyzes oscillations in the absorption spectrum that occur tens to hundreds of eV above the absorption edge [114]. These oscillations arise from interference patterns of the photoelectron wave scattered by neighboring atoms, providing details on local atomic arrangements such as bond lengths, coordination numbers, and the identity of the elements surrounding the absorbing atom [114]. Each scattering path between atoms gives a distinct peak in the EXAFS spectrum and can be used to identify the existence of single atoms. For highly dispersed metal oxide species, metal-oxygen contributions will be primarily seen in the spectra because oxygen atoms (as well as other elements from the support) will typically be the nearest neighbors of the absorbing atom, and they comprise the “first shell” [114]. The next-nearest neighbors comprise the “second shell”, and it should include the same contributions as the first shell if single atoms are the case. If strong metal–metal contributions arise from either shell, it indicates that metal atoms are too close (suggesting below 5 Å), which is the effective range of the EXAFS spectra) and are probably forming clusters.

Although extensively applied, XAS methods have several notable drawbacks apart from the high cost mentioned in the following table. Firstly, the high-intensity synchrotron beams essential for detection can inflict “beam damage”, particularly if samples contain minimal amounts of the element in question or if measurements must be taken under in situ/operando conditions [115,116,117]. Prolonged exposure may artificially modify oxidation states or induce clustering, thereby complicating accurate data interpretation. Secondly, EXAFS does not directly yield a single atom structural model. Instead, interpreting its data requires fitting to proposed structural frameworks, making outcomes heavily dependent on the chosen assumptions. Lastly, EXAFS is inherently an averaging method, so all atoms of a given element contribute to the final signal. If a sample contains multiple local configurations, distinctions crucial to single atom behavior might be masked [114,116]. Therefore, corroborating EXAFS findings with additional analytical techniques, alongside control experiments to account for beam effects, is strongly recommended for reliable characterization.

*X-ray Photoelectron Spectroscopy*: XPS is a surface-sensitive analytical technique that measures the binding energies of core-level electrons by exciting them to the point they escape the atom and the material [118]. As a result, it provides insight into the chemical states and electronic environments of elements present at or near a material’s surface. By detecting subtle shifts in binding energy and changes in peak intensity or shape, XPS can distinguish between different oxidation states, coordination environments, and the influence of support interactions on atomic species [89]. When applied to the study of single atom catalysts, these principles are particularly useful. Isolated atoms anchored on support often exhibit unique electronic signatures compared to atoms in clusters or bulk materials [99]. The altered electron density and modified chemical bonding at the scale lead to characteristic binding energy shifts and distinct spectral features in XPS data [99]. Through careful analysis of these changes and with comparison to known reference states, XPS can assist in confirming the presence of single atom sites.

Despite its usefulness, XPS only probes a shallow depth (~1 nm), so it may struggle with very low loadings of SA. Additionally, beam-induced transformations can occur, as mentioned in Table 3, such as the reduction of Pt (IV) to Pt (II), sometimes in a matter of minutes [119,120]. Careful experimental design—like minimizing exposure times or using filters—is crucial to mitigate damage. Furthermore, accurate spectral fitting is essential; improper calibration, handling of spin–orbit coupling, and peak deconvolution can lead to incorrect conclusions. Consequently, while XPS is a key tool for identifying single atom sites, caution must be exercised to ensure reliable data.

*Electron Paramagnetic Resonance*: EPR spectroscopy is a technique applicable to paramagnetic species, as mentioned in Table 3, that utilizes the Zeeman effect on unpaired electrons and measures their microwave absorbance as they transit between energy states [121]. More specifically, as the magnetic field is scanned in a selected range, unpaired electron spins in the sample absorb energy at specific resonant frequencies, producing characteristic EPR signals [121]. These signals are influenced by factors such as the electronic environment, the nature of neighboring atoms, and the symmetry of the local coordination site. For single atom catalysts, if the individual metal atoms possess unpaired electrons (as is often the case for certain transition metals like Cu) [122], their EPR spectra may exhibit distinct signatures that differ substantially from those of clusters or bulk phases [96], which tend to have more complex and broadened EPR responses. By comparing the EPR parameters such as g-values, hyperfine splittings, and line shapes with known references and simulated data, one can conclude the presence of isolated atoms [121]. Thus, EPR can be a valuable tool for confirming single atom confinement of paramagnetic species [123].

Carbon Monoxide Diffuse Reflective Infrared Fourier Transform Spectroscopy (CO-DRIFTS) uses CO as a probe molecule to identify and characterize metal or metal-oxide sites by tracking CO’s vibrational signals upon adsorption [124,125]. In practice, CO interacts with different surface sites—such as isolated atoms, nanoclusters, or bulk phases—yielding distinct IR peaks (e.g., linear and bridging) that reflect variations in bonding environments. This makes CO-DRIFTS especially valuable for detecting single atoms or nanoclusters anchored on supports, as linear CO often shows sharp, narrow peaks indicative of isolated sites. For instance, B. Han et al. utilized CO-FTIR to distinguish between single atoms and nanoparticles anchored on TiO_2_ [17]. Nonetheless, the method has certain drawbacks. For instance, surface sites that do not bind CO strongly may remain undetected, and overlapping signals from other metal oxides or structural vibrations can complicate data interpretation. Moreover, low metal loadings or highly heterogeneous samples may produce weak signals, affecting sensitivity [124]. Despite these limitations, with careful experimental design—such as selecting appropriate backgrounds and pretreatment protocols—CO-DRIFTS remains a robust tool for examining metal/metal oxide surfaces at the single atom or nanocluster level [126].

In addition to experimental detection methods, theoretical tools such as Density Functional Theory (DFT), a quantum mechanical modeling method, can be used to investigate the electronic structure of many-body systems, primarily atoms, molecules, and solids [127]. Reviewing the theoretical methods is out of the scope of the present review; however, the interested reader can consult pertinent review articles on DFT simulations on ad atoms on metal oxides [128,129,130]. When applied to single atom catalysts, DFT calculations can predict how isolated atoms interact with the support, estimate binding energies, forecast reaction pathways, and simulate spectroscopic signatures (for example, shifts in core-level energies or changes in vibrational frequencies). DFT simulation may also extend to catalytic performances [109]. If the simulated data align with the experimental results, they can serve as solid proof for single atoms’ existence [95].

## 4. Single Atoms/Sub-Nanoclusters and Quantum-Sized Small Particles of Co-Catalysts on Perovskite Oxide and Non-Perovskite Oxide Substrates

In this section, we discuss cases of SA/SNC and/or QSSPs deposited on perovskite oxide and non-perovskite oxide substratesreporting the synthetic routes and emphasizing the characterization techniques used to identify the exact configuration of the co-catalyst, in addition to their performance on specific photocatalytic applications.

### 4.1. Single Atoms/Sub-Nanoclusters and Quantum-Sized Small Particles of Co-Catalysts on Perovskite Oxide

**Figure 6:**
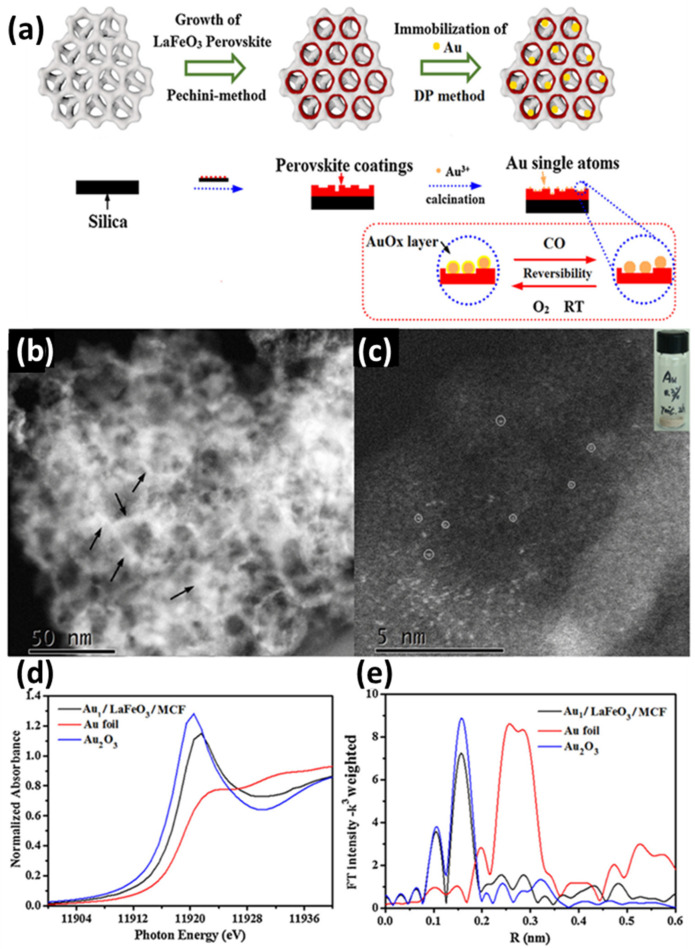
(**a**) Schematic illustration of the Au_1_/LaFeO_3_/MCF preparation process; (**b**) STEM image (the black arrows highlight the region of the HAADF-STEM images) and (**c**) HAADF-STEM image of Au_1_/LaFeO_3_/MCF (the white circles marks the Au single atoms); (**d**) X-ray absorption near edge spectrum (XANES) of Au_1_/LaFeO_3_/MCF, Au foil, and Au_2_O_3_; (**e**) and Au LIII-edge EXAFS of Au_1_/LaFeO_3_/MCF, Au foil, and Au_2_O_3_. Reprinted from [68], with permission from Elsevier.

C. Tian et al. reported [68] the synthesis of a hierarchical LaFeO_3_ perovskite support decorated with stable single Au atoms. The synthesis of the substrate LaFeO_3_/MCF was based on the Pechini method [131], and the Au species was later incorporated through a deposition–precipitation process employing HAuCl_4_ in water, achieving an approximate loading of 0.3 wt.%. The final structure was obtained by calcinating the Au-loaded LaFeO_3_/MCF pre-catalysts in air at 700 °C for 2 h. To examine the dispersed state of the Au species, HAADF-STEM was utilized [68]. As anticipated, numerous randomly distributed atomic Au species were identified, marked by white circles (see Figure 6c). The atomically dispersed individual Au atoms were further verified via XAFS spectroscopy. Additionally, according to the XANES of Au_1_/LaFeO_3_/MCF, Au foil, and Au_2_O_3_, Au_1_/LaFeO_3_/MCF exhibits a strong peak around 11,921 eV (see Figure 6d [68]), which corresponds to the depletion of Au d orbitals from their metallic d10 configuration. This indicates that the Au species supported on the perovskite exhibit notable oxidic characteristics, similar to the Au^3+^ in Au_2_O_3_. DFT calculations were performed to confirm the presence of oxidized Au species on the LaFeO_3_ surface [68]. Overall, Au adsorption on LaFeO_3_ surfaces was found to be stronger than on TiO_2_, aligning with the observed greater resistance to sintering, proving that LaFeO_3_ perovskite is a better substrate to noble metals than TiO_2_ metal oxide [68]. The Au_1_/LaFeO_3_/MCF catalyst was evaluated by its catalytic activity toward CO oxidation. By leveraging the strong interaction between gold atoms and the LaFeO_3_ perovskite support, this system achieves exceptional stability and activity under reaction conditions. The synergistic combination of LaFeO_3_’s structural and electronic properties with the catalytic efficiency of gold single atoms enables highly selective and efficient photocatalytic performance, even under challenging conditions [68].

H. Shin and coworkers [91] reported the deposition of Pt single atoms on different supports, metallic, metal oxide, and perovskite nanosheets, reaching up to 3.94 wt.% Pt loading in the case of perovskite LaCoO_3_. The concept of the synthesis involved the use of N-doped graphene as a sacrificial template to locally constrain the single atoms [91]. The process includes adsorbing precursors of the desired support material onto the SA-stabilized template, followed by heat treatment to transfer the SAs onto the support material while removing the graphene layer [91]. By controlling the amount of the Pt during the synthesis, Pt single atoms (3.94 wt.% Pt) and Pt clusters (12.7 wt.% Pt) were formed on the LaCoO_3_ perovskite. HAADF-STEM analysis was performed on Pt-SAs/LaCoO_3_ and PtCl/LaCoO_3_ [91]. In the first case, Pt species were uniformly dispersed and atomically distributed across the perovskite’s surface, while in the latter, Pt clusters and small nanoparticles were observed. To further identify and compare single atoms and nanoclusters on LaCoO_3_, EXAFS analysis was utilized, confirming that in Pt-SAs/LaCoO_3_ [91] spectra, no Pt-Pt bond contribution is resolvable, while in PtCl/LaCoO_3_, the Pt-Pt bond appears, verifying the presence of clusters or small particles [91].

Djatoubai et al. [93] used Co single atoms to decorate the surface of BiFeO_3_ perovskite. The synthesis involved a facile hydrothermal method to form the perovskite and an immersion Wet chemistry method to deposit the Co species. The deposition of Co-SAs was successfully confirmed using aberration-corrected high-angle annular dark-field scanning transmission electron microscopy (AC HAADF-STEM) and X-ray Absorption Near-Edge Structure (XANES) [93]. In the EDS spectrum, the homogeneous Co distribution on BiFeO_3_ was verified, while the high-resolution X-ray photoelectron spectroscopy confirmed the presence of Co^2+^ species. The photocatalytic system achieved a great O_2_ evolution response at solar water oxidation reactions, surpassing BiFeO_3_-based materials. The performance was attributed to the Co-SAs utilization, which enhanced charge-carrier separation and electron transport [93].

Y. Xing et al. [87] reported the synthesis of SrTiO_3_ perovskite decorated with either single atoms or large clusters and nanoparticles of Pt. The formation of Pt large clusters and nanoparticles on the substrate’s surface (Pt/SrTiO_3_) was conducted using an impregnation method, while the catalyst with the Pt single atoms was prepared by leaching the Pt/SrTiO_3_ catalyst (Pt/SrTiO_3_-L) [87]. The aggregation state of the Pt species was studied with High-Resolution STEM images (HR-STEM). In the case of Pt/SrTiO_3_ catalyst, Pt nanoparticles were observed and measured at 2.7 nm in size, while the image of Pt/SrTiO_3_-L catalyst single atomic Pt and small Pt clusters was detected [87], composed of 2–8 atoms. Based on the extended EXAFS results, unlike the standard Pt foil and PtO_2_ samples, no significant Pt–Pt bonds were detected in either of the catalysts [87]. In the Pt/SrTiO_3_-L catalyst, the peak at 1.58 Å, corresponding to Pt–O coordination, was sharper compared to that of Pt/SrTiO_3_. Additionally, the second peak observed at 2.8–3.2 Å, associated with PtO_2_ in the Pt/SrTiO_3_-L catalyst, deviates from the reference, indicating that the Pt species are predominantly sub-nanoscale and exist in a highly undercoordinated state [87]. The catalysts with different aggregation states of co-catalyst were evaluated via reverse water gas shift reactions, leading to the conclusion that the catalyst decorated with Pt single atoms/nanoclusters demonstrates higher CO_2_ conversion compared to the one with the large Pt clusters/nanoparticles [87]. The size effects of Pt in the RWGS reaction were studied using DRIFTs and DFT methods. Single Pt atoms/Sub-nanoclusters showed the highest activity and CO selectivity via the “–COOH route”, [87] whereas larger Pt particles promoted further CO hydrogenation. Over Pt/SrTiO_3_, the rate-determining step is the reaction between formate and H*, which can be hindered by the strong formate adsorption observed on large Pt clusters and nanoparticles [87].

Z. Wang et al. [92] studied the decoration of Ni atoms on SrTiO_3_. In their study, Ni atoms, as well as clusters, were reportedly deposited on the SrTiO_3_ (100) surface. The perovskite material was purchased readily in the form of single crystals (5 mm × 5 mm × 5 mm), doped with Nb 5 wt.% [92]. The Ni deposition was carried out through the e-beam evaporation method and produced two samples of different Ni coverage. The first sample had 0.01 Å of Ni coverage (an assumption based on the vapor deposition rate), and the second sample had 0.05 Å of Ni coverage on its surface [92]. Scanning transmission electron microscopy (STEM) experiments were carried out for these samples. For the first sample, the results revealed Sr adatoms, as well as two types of bright protuberances that were labeled as Ni atoms because of their adsorption site and their height of approximately 150 pm [92]. Three orientation models were examined using DFT calculations [92] and were proposed as the most energetically favorable over all other possible orientations. Regarding the second sample, Ni sites were observed at 300–400 pm height and were attributed to cluster formation. Valence-band photoelectron spectroscopy (VB-PES) experiments were later conducted and found new in-gap states on different energy levels for the two samples. While the techniques mentioned within this paragraph were used thoroughly, they cannot be considered adequate for precisely distinguishing the configuration of the ad atoms. As stated in previous sections, it is most possible that the results from an atom anchoring process follow a distribution that ranges from single atoms to nanoclusters and nanoparticles. STEM imaging can give a clear enough image but of local range, and more characterization techniques should be utilized to conclude this study [92].

**Figure 7:**
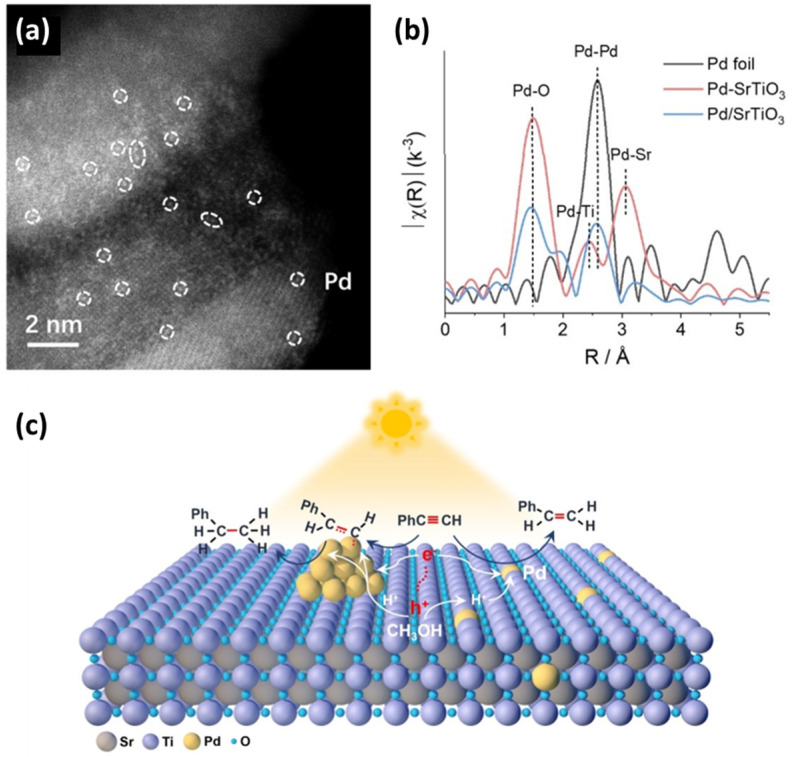
(**a**) AC HAADF-STEM images of Pd-SrTiO_3_, with single atoms of Pd highlighted in white circles; (**b**) Pd K-edge EXAFS spectra of Pd foil, Pd-SrTiO_3_, and Pd/SrTiO_3_; (**c**) possible reaction mechanism of PA hydrogenation reaction over Pd-SrTiO_3_ and Pd/SrTiO_3_. Reproduced with permission from ref. [94]. Copyright {2024} Wiley-VCH.

A study on Pd-SrTiO_3_ by B. Yang et al. [94] does not report single atom deposition on the perovskite’s surface but rather single atom doping and decorating clusters [94] based on several pertinent characterization techniques (see Figure 7). Specifically in [94], two SrTiO_3_ samples were synthesized using a solvothermal method, and Pd introduction was created by means of an aqueous solution. There were several loading amounts ranging from 0.1 to 0.8 wt.%. The first sample was labeled as Pd-SrTiO_3_, and it would later be characterized as SrTiO_3_ with single atoms inside the lattice, and the second sample was labeled as Pd/SrTiO_3_, and it would later be characterized as Pd cluster-decorated SrTiO_3_. Regarding Pd-SrTiO_3_, AC HAADF-STEM (see Figure 7a) made a clear demonstration of highly dispersed Pd single atoms inside the perovskite lattice [94]. The existence of single atoms in the lattice environment was further confirmed by Pd K-edge EXAFS (see Figure 7b [94]). The same techniques were applied to the Pd/SrTiO_3_ sample and proved the existence of clusters through the observed Pd-Pd bonds [94]. The results were compared to Pd-foil for reference [94]. Several other techniques, including FTIR, Temperature-Programmed Desorption (TPD), EPR, and DFT, were used for the indirect identification of the single atoms and the clusters, as well as for the evaluation of the catalysts’ performance. The photocatalytic semi-hydrogenation of alkynes experiments demonstrated that the incorporation of Pd single atoms within the SrTiO_3_ lattice [94] (see Figure 7c), as opposed to being supported on its surface, effectively altered the adsorption behavior of phenylacetylene (PA) and styrene on the photocatalysts. Pd-SrTiO_3_, exhibiting a sufficiently strong interaction with PA but a relatively weak interaction with styrene, is expected to offer significant advantages in the photocatalytic semi-hydrogenation of PA, such as achieving high yield with excellent selectivity for styrene [94].

The use of SrTiO_3_ as support was also studied by J. Fend et al. [95] for decoration with Au single atoms. The team utilized commercially available SrTiO_3_ doped with 7 wt.% Nb and applied a thermal evaporation method for Au atom deposition. The sample preparation involved three steps: first, the deposition of Ti atoms to "reconstruct" the SrTiO_3_ surface, followed by the sputtering of Au^+^ ions, and, finally, calcination at high temperature to stabilize the structure [95]. The characterization part of this study only included scanning transmission electron microscopy equipped with Molecular Beam Epitaxy (MBE), Reflection High Energy Electron Diffraction (RHEED), and Low Energy Electron Diffraction (LEED) [95]. According to STEM images, bright and dark spots appear, while probably based on the Z factor previously discussed, the team attributed the bright spots to Au atoms [95]; however, these could also be small Au clusters since STAM cannot provide such resolution. They then compared the number of bright spots to the amount of Au loading to deduce that each bright spot contains only one Au atom.

Our group recently reported the synthesis of NaTaO_3_ perovskite oxide and simultaneously the deposition of ultrafine NiO nanoparticles using one-step flame spray pyrolysis technology [74]. By utilizing the Double Nozzle setup, two different nanocrystals were formed, leading to a tightly interfaced heterojunction. The deposited NiO nanoparticle’s size was calculated at an average size of 2 nm using TEM, XRD, and BET characterization techniques. Various Ni loadings were deposited on the NaTaO_3_ surface, and according to the H_2_ production evaluation, lower Ni content (0.5 wt.%) was optimal since, in the case of high Ni loadings, the coverage of NaTaO_3_ surface is higher, which leads to the inhibition of photoexcitation [74].

### 4.2. Single Atom/Sub-Nanoclusters and Quantum-Sized Small Particles of Co-Catalyst on Non-Perovskite Oxide

**Figure 8:**
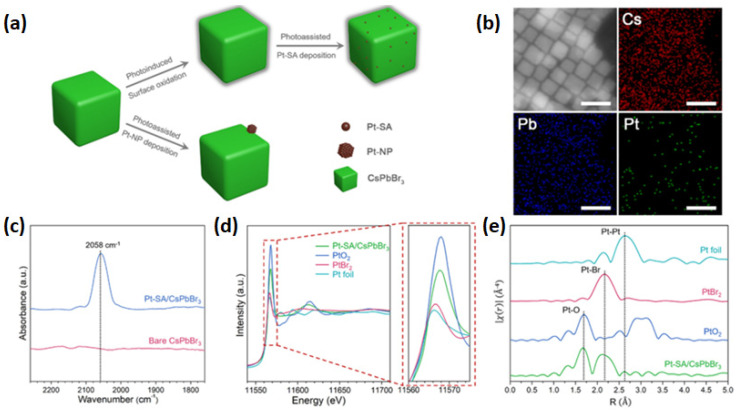
(**a**) Schematic illustration of the formation mechanism of Pt-SAs/CsPbBr_3_ and Pt-NPs/CsPbBr_3_ and (**b**) HAADF-STEM image and EDS mapping of 1.04% Pt-SAs/CsPbBr_3_ nanocrystals. The scale bars are 20 nm. (**c**) DRIFT spectra of CO adsorption on bare CsPbBr_3_ nanocrystals and 1.04% Pt-SAs/CsPbBr_3_ nanocrystals. (**d**) Normalized XANES spectra at the Pt L3-edge of Pt-SAs/CsPbBr_3_, PtO_2_, PtBr_2_, and Pt foil. (**e**) Normalized k3-weighted Fourier transform spectra derived from EXAFS of 1.04% Pt-SAs/CsPbBr_3_, PtO_2_, PtBr_2_, and Pt foil. Reprinted (adapted) with permission from [89]. Copyright {2021} American Chemical Society.

Hu et al. [89] reported that Pt single atoms can be deposited on the surface of CsPbBr_3_ nanocrystals using a photo-assisted method (see Figure 8a). This process involves partial oxidation of the surface, followed by anchoring Pt single atoms through the formation of Pt–O and Pt–Br bonds, resulting in a 1.04 wt.% loading amount of Pt. In the case of the non-oxidized surface, the formation of Pt nanoparticles is favorable since the metal–halide bind cannot efficiently stabilize the highly active Pt single atoms [89]. To verify the atomic deposition of single Pt atoms onto CsPbBr_3_ nanocrystals, energy-dispersive X-ray spectroscopy (EDS) (see Figure 8b) was performed, confirming that the Pt atoms were homogeneously dispersed on the perovskite’s surface [89]. Diffuse reflectance infrared Fourier transform (DRIFT) spectroscopy was conducted using CO as a probe molecule to investigate the form of Pt in Pt-SAs/CsPbBr_3_. In the CO-DRIFTS spectrum of Pt-SAs/CsPbBr_3_ nanocrystals, a prominent peak appears around 2058 cm^−1^, corresponding to the linear adsorption of CO on Pt^δ+^ sites [89]. The absence of a CO bridge adsorption peak, typically associated with two Pt atoms, suggests that the Pt atoms remain isolated without forming Pt nanoparticles or experiencing significant agglomeration [89]. Additionally, according to the normalized XANES spectrum, the shape of Pt/CsPbBr_3_ differs from the other spectra, such as Pt foil, PtO_2_, and PtBr_2_, indicating a different electronic structure (see Figure 8d). The spectrum for the as-prepared 1.04% Pt-SAs/CsPbBr_3_ reveals two prominent features at approximately 1.7 Å and 2.2 Å, corresponding to Pt–O and Pt–Br scattering paths, respectively. Notably, no signal near 2.6 Å is detected, indicating the absence of Pt–Pt scattering paths [89], as observed in the reference Pt foil. These EXAFS findings confirm that Pt atoms are well-dispersed on the CsPbBr_3_ surface, forming Pt–O and Pt–Br bonds [89]. According to their photocatalytic performance in the semi-hydrogenation of propyne, Pt-SAs/CsPbBr_3_ displayed high quenching efficiency, which the researchers assigned to the formation of deep trap sites after Pt single atom deposition, resulting in fast transfer carrier from perovskite to Pt active sites during illumination [89].

**Figure 9:**
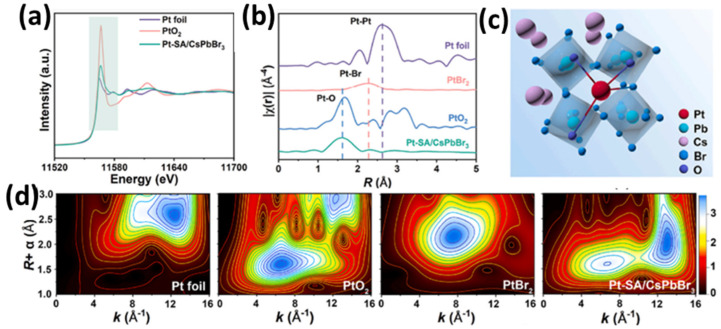
(**a**) XANES spectra for Pt K-edge of Pt foil, PtO_2_, and Pt-SAs/CsPbBr_3_. (**b**) Fourier transform K-edge EXAFS spectra and (**d**) wavelet transforms of the k3-weighted EXAFS signals of Pt foil, PtO_2_, PtBr_2_, and Pt-SAs/CsPbBr_3_ and (**c**) schematic structural model of Pt-SAs/CsPbBr_3_. Reprinted from [86], with permission from Elsevier.

Another work on single Pt atom deposition on CsPbBr_3_ nanocrystals was provided by Y. Qin and coworkers [86]. The Pt/CsPbBr_3_ nanocrystals were prepared using the two-step wet chemistry method. First, the surface of the perovskite was partially oxidized via light irradiation in order to form Pb-O bonds. These active sites can effectively anchor and stabilize Pt single atoms through coordination with Br atoms. After Pt deposition, to avoid possible aggregates due to high temperature, a deuterium lamp was utilized at room temperature to stabilize the Pt single atoms on the perovskite substrate. The EXAFS profiles (see Figure 9d) [86] revealed that the Pt species were in an oxidized state, while from the EXAFS spectra in R space, it was found that except Pt-O and Pt-Br bonds, no other peaks assigned to Pt-Pt were observed (see Figure 9d). As concluded, Pt atoms are highly dispersed and isolated on CsPbBr_3_ perovskite. The Pt single atom/CsPbBr_3_ nanocrystals were evaluated to determine their electrocatalytic performance in the detection of ascorbic acid, reaching the detection limit of 0.0369 mM. Pt single atoms served as quantitative active sites for substrate adsorption and facilitated charge transfer by efficiently capturing electrons for electrochemical reactions [86].

Based on a hydrothermal method followed by an impregnation method, P. Zhou et al. [88] reported strong anchoring of highly dispersed Pt single atoms on the surface of Cs_2_SnI_6_ perovskite. The conventional low-temperature and rapid crystallization methods, such as hydrothermal methods commonly used for synthesizing perovskites, inevitably result in the introduction of numerous defects into the perovskite structure [88]. As mentioned before, these defects on the substrate enhance the strong anchoring of the metal co-catalyst on the catalyst’s surface, resulting in strong metal–support interaction (SMSI). The loading of Pt single atoms on the substrate was calculated to be 0.12 wt.%. To confirm the formation of single Pt atoms on the perovskite’s surface, HAADF-STEM was employed [88]. According to the HAADF-STEM image and EDS mapping images presented, there is a uniform distribution of Pt single atoms on Cs_2_SnI_6_, while no Pt clusters or nanoparticles were observed [88]. Furthermore, the oxidation state and coordination environment of Pt single atoms were examined via XAFS. Based on the normalized Pt L3-edge XANES spectra for Pt-SAs/Cs_2_SnI_6_, PtI_2_, and Pt foil, it was found that the oxidation state of Pt in Pt-SAs/Cs_2_SnI_6_ exceeds +2, while the FT- EXAFS spectra [88] indicate that Pt atoms are anchored to the surface of PtSAs/Cs_2_SnI_6_ via a Pt–I bond. Pt-SAs/Cs_2_SnI_6_ was evaluated for photocatalytic hydrogen production in comparison to Cs_2_SnI_6_ decorated with Pt nanoparticles and pristine Cs_2_SnI_6_, resulting in an enhanced H_2_ evolution rate [88] for Pt-Cs_2_SnI_6_. Through a combination of charge-carrier dynamics studies and DFT calculations, we identified that the distinctive coordination structure and electronic properties of Pt–I_3_ species play a crucial role in the SMSI effect. This enhances photogenerated electron transfer from Cs_2_SnI_6_ to Pt single atoms while simultaneously lowering the Gibbs free energy and accelerating the kinetics of hydrogen production [88].

**Figure 10:**
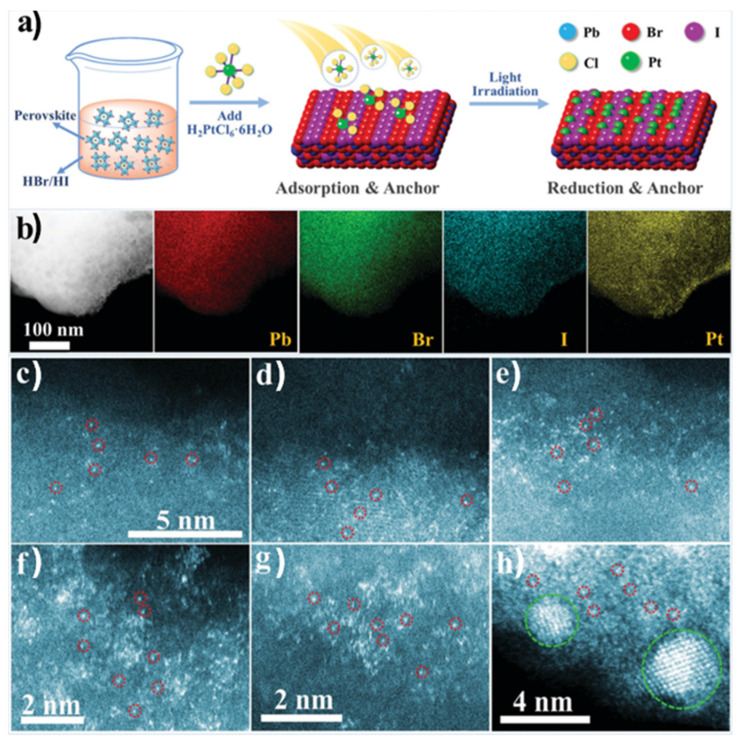
Synthesis mechanism illustration and STEM-HAADF images of Pt/FAPbBr_3−x_I_x_. (**a**) Mechanism illustration of the synthesis process of Pt/FAPbBr_3−x_I_x_. (**b**) TEM images and corresponding EDS mapping images of a random area of Pt/FAPbBr_3−x_Ix. STEM-HAADF images of Pt/FAPbBr_3−x_I_x_ with different Pt loading amounts of (**c**), 0.7; (**d**), 1.2; (**e**), 1.5; (**f**), 1.8; (**g**,**h**), 2.1. (**d**,**e**) share the same scale bar with (**c**). Some of the Pt atoms are circled in red in (**c**–**h**), and Pt clusters are circled in green in (**h**). Reproduced from Ref. [90] with permission from the Royal Society of Chemistry.

A different approach was followed by Y. Wu et al., employing an organometal halide perovskite as a substrate for single atoms Pt deposition [90] (see Figure 10a). The synthesis of FAPbBr_3−x_I_x_ (FA = CH(NH_2_)_2_) particles involved a wet chemistry co-precipitation method, while for the Pt deposition on the surface of the perovskite, the Pt species were absorbed and reduced under light irradiation. Different Pt loadings were performed from 0.7 to 2.1 wt.% on the substate’s surface in order to explore the limits in which the Pt single atoms aggregate to nanoclusters and eventually to nanoparticles. The catalysts with different Pt loadings were evaluated by STEM-HAADF and EDS (see Figure 10b). The images of elemental mapping proved the existence of the Pt element on the perovskite and its uniform distribution. The HAADF images confirmed that as the Pt loading increases above 1.8 wt.%, Pt nanoclusters and Pt nanoparticles are formed. The researchers further used XPS and XAFS measurements [90], which revealed that the Pt species exist in distinct chemical environments, likely due to variations in their coordination with Br or I. Additionally, DFT was employed to develop the theoretical model, demonstrating that halide ions on the surface of the mixed-halide FAPbBr_3−x_I_x_ perovskite are well-suited to serve as uniform anchoring sites for Pt atoms. The catalysts with different co-catalyst loadings were examined by photocatalytic hydrogen production experiments, demonstrating that the optimal Pt loading was 1.8 wt.%, which, based on the former analysis, corresponds to single Pt atoms dispersed on the perovskite surface [90].

### 4.3. Single Atoms/Sub-Nanoclusters and Quantum-Sized Small Particles (QSSPs) of Co-Catalyst on Metal Oxides

For completeness, we include a few examples of popular metal oxides, such as TiO_2_ and ZrO_2_, to discuss and further highlight the controversies in the literature regarding the identification of the exact aggregation state of the co-catalyst on the substrate.

Y. Zhang et al. reported the dispersion of a high amount of Cu single atoms (~1.5 wt.%) on the surface of a TiO_2_ semiconductor. Utilizing a metal–organic framework (MOF), the metal ions were anchored into the MOF by a wet impregnation method, forming a metal–oxygen–titanium bond, followed by thermal annealing. HAADF-STEM was employed to verify that the Cu atoms, shown by the bright spots, were incorporated on the Ti vacancy sites in the intermediate MOF [96]. The line scan profiles, highlighted with three randomly selected blue lines, show that line 1 consists solely of Ti atoms, whereas lines 2 and 3 contain both Ti and Cu atoms, confirming the presence of Cu-O-Ti clusters [96]. The valence states of Cu in CuSA-TiO_2_ were examined by in situ Electron Paramagnetic Resonance (EPR) both before and after irradiation. Prior to irradiation, the EPR spectrum of CuSA-TiO_2_ exhibited a prominent Cu^2+^ signal, which diminished following 30 min of irradiation but subsequently intensified upon air exposure. This observation suggests that Cu^+^ species, which are EPR-silent, were generated during irradiation and later oxidized back to Cu^2+^ upon contact with air [96]. Although the concept of this work was based on the enhanced photocatalytic properties of Cu single atoms on TiO_2_, the EPR signal shape prior to the illumination reveals the coexistence of Cu sub-nanoclusters alongside Cu single atoms. CuSA/TiO_2_ with 1.5 wt.% Cu semiconductor showed excellent photocatalytic hydrogen production compared to the catalysts decorated with other Cu loadings (from 0.48 to 2.57 wt.%), while the overall enhanced performance of the system was attributed to the efficient electron transfer through the Cu^2+^–Cu^+^ process.

**Figure 11:**
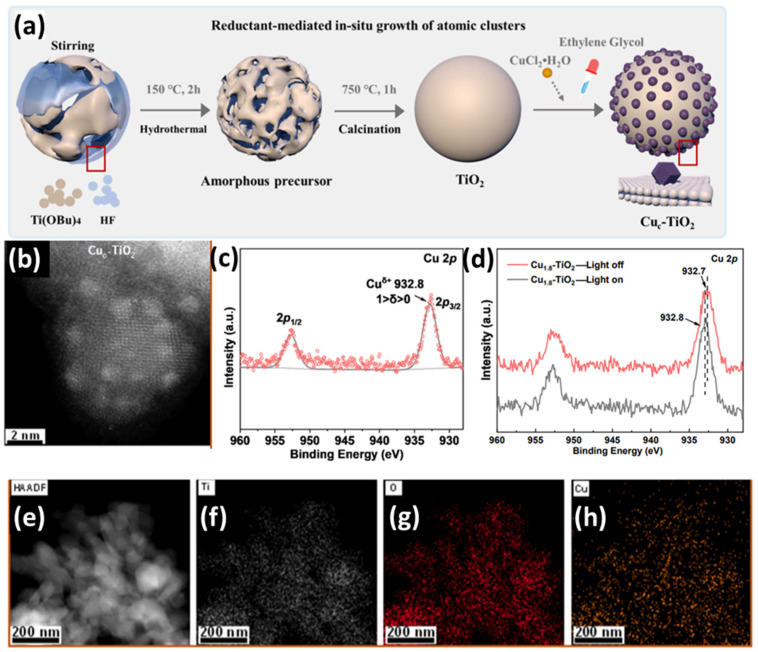
(**a**) Schematic illustration of the specific synthesis process of Cu_c_-TiO_2_; (**b**) atomic resolution HAADF-STEM image of Cu_1.8_-TiO_2_, (**c**) high-resolution XPS analysis of Cu 2p, (**d**) in situ XPS Cu 2p spectra before and after irradiation, (**e**–**h**) HAADF-STEM image and EDX mapping of Cu_1.8_-TiO_2_. Reprinted (adapted) with permission from [97]. Copyright {2024} American Chemical Society.

H. Zou and coworkers [97] developed a TiO_2_ semiconductor decorated with Cu sub-nanoclusters, achieving a metal mass loading of 1.8 wt.% (see Figure 11a). The TiO_2_ substrate was prepared via a hydrothermal method followed by calcination, while the Cu deposition occurred via a wet impregnation method. According to XRD patterns, no metallic diffraction peak of Cu or CuO NPs was observed [97], suggesting that Cu is in the aggregation state of nanoclusters. Furthermore, aberration-corrected STEM was employed, and Z-contrast HAADF images (see Figure 11b) demonstrated the presence of ultrafine Cu sub-nanoclusters (approximately 1 nm in diameter), distributed on the semiconductor’s surface [97]. The chemical states of Cu were investigated by XPS (see Figure 11c,d). The observed negative shifts in the Ti 2p and O 2p peaks upon introducing Cu, along with the positively charged valence states of the metals, indicate significant electron transfer from the metal co-catalysts to TiO_2_ [97].

Moreover, the Cu 2p peaks confirm the successful integration of Cu clusters, emphasizing strong charge interactions with the substrate. The TiO_2_ decorated with Cu subnanoclusters exhibited remarkable hydrogen production, which indicates that the decoration of Cu clusters enhances both light absorption and the transport and separation of electron–hole pairs while also reducing photogenerated charge recombination. Furthermore, DFT calculations suggested that the surface-active, site-rich SNC-Cu exhibits favorable hydrogen adsorption and desorption energy barriers [97].

**Figure 12:**
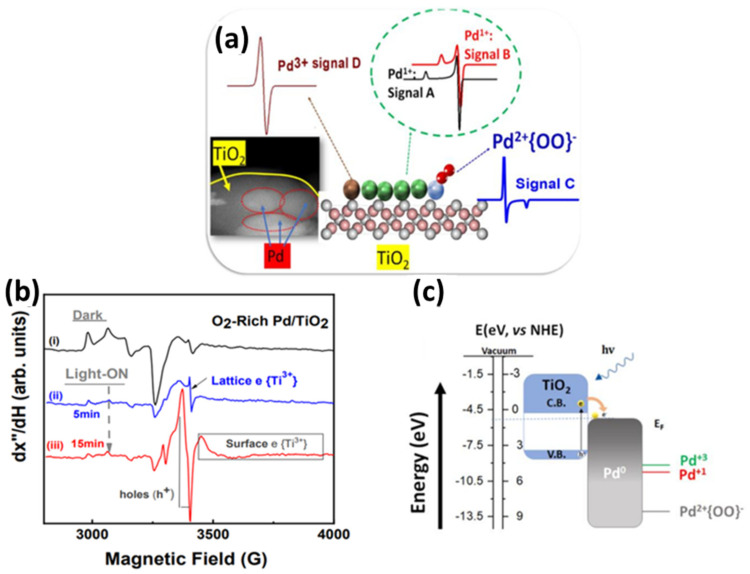
(**a**) Schematic depiction of structural assignment of the detected Pd^n+^ EPR signals. The STEM image shows the Pd particles deposited on the TiO_2_ matrix. (**b**) 77K EPR spectra of O_2_-Rich Pd/TiO_2_ illuminated with a Xenon solar simulator at different illumination times. (**c**) Schematic photoinduced electron transfer mechanism under photocatalytic conditions. Reprinted (adapted) with permission from [71]. Copyright {2022} American Chemical Society.

Our group recently reported a library of atomic Pd states formed on Pd-Nps, stabilized on TiO_2_ support, using flame spray pyrolysis (FSP) technology [71]. Our findings revealed that interfacial Pd atoms can stabilize in Pd^1+^ or Pd^3+^ oxidation states depending on the oxygen availability and local temperature at the Pd-TiO_2_ interface during FSP synthesis. Various post-FSP treatments were conducted, such as reduction with gas H_2_, treatment with NaHB_4_, and oxidation under O_2_. Moreover, these systems were evaluated under formic acid dehydrogenation catalytic conditions and via in situ photocatalytic EPR study [71]. Consequently, through EPR spectroscopy, we identified four distinct Pd states at the Pd/TiO_2_ interface, presented in Figure 12a: Pd^0^, Pd^1+^, {Pd^2+^–O_2_^−^}, and Pd^3+^. The Pd^0^ species were observed under oxygen-lean preparation conditions or in highly reducing environments, such as hydrogen treatment without H_2_O, while the formation of the less common Pd^1+^ and Pd^3+^ states was found to be directly linked to the abundance of oxygen [71]. Although Pd^2+^ species are not visible by EPR spectroscopy, a unique {Pd^2+^–OO^−^} state appeared under specific conditions, including H_2_/H_2_O treatment or during catalytic formic acid (HCOOH) dehydrogenation. The case of the O_2_-rich Pd/TiO_2_ sample shows a highly efficient transfer of photogenerated electrons to Pd-Nps, which prevents electron–hole recombination, thus enhancing photocatalytic performance [71] (Table 4).

**Table 4:** Literature summary of perovskites and metal-oxide substrates decorated with single atoms/sub-nanoclusters/quantum-sized small particles of various metals and their application in photocatalysis.

Nanostructure	Co-Catalyst	Aggregation State of the Co-Catalyst	Method of Analysis	Catalytic Application	Ref.
Perovskites					
LaFeO_3_	Au	SACs	HAADF-STEM, EDS, XAFS, DFT, EXAFS, XANES	CO oxidation	[68]
SrBO_3_ (B = 3d transition metals)	Pt	SACs	DFT	Methane activation	[109]
CsPbBr_3_	Pt	SACs, NPs	HR-TEM, 3D-FP XANES, EXAFS	Photocatalytic semi-hydrogenation of propyne	[89]
SrTiO_3_	Pt	SACs, SNCs, NPs	HAADF-STEM, EXAFS	Reverse water–gas shift reactions	[87]
Cs_2_SnI_6_	Pt	SACs, NPs	HAADF-STEM, EDS, XAFS, XPS, EXAFS, EXANS, DFT	Photocatalytic hydrogen production	[88]
CsPbBr_3_	Pt	SACs	HAADF-STEM, EDS, XPS, DRIFT, EXAFS, XANES	Electrochemical sensing of ascorbic acid	[86]
FAPbBr_3−x_I_x_ (FA = CH(NH_2_)_2_)	Pt	SACs	HAADF-STEM, EDS, XPS, DFT, EXAFS, XANES	Photocatalytic hydrogen production	[90]
LaCoO_3_	Pt	SACs, SNCs	HAADF-STEM, EDS, EXAFS, EPR, XANES, XPS	Chemiresistive sensing of acetone gas	[91]
NaTaO_3_	Ni	NPs	TEM, XRD, BET	Photocatalytic hydrogen production	[73]
SrTiO_3_	Ni	SACs, SNCs	STEM, PES, DFT	-	[92]
BiFeO_3_	Co	SACs	AC HAADF-STEM, EDX, XPS, XANES, EXAFS	Photocatalytic oxygen evolution	[93]
SrTiO_3_	Pd	SACs, SNCs	AC HAADF-STEM, XAS, XPS, XANES	Photocatalytic semihydrogenation of Alkynes	[94]
SrTiO_3_	Au	SACs	STEM-MBE/RHEED/LEED	-	[95]
Metal Oxides					
TiO_2_	Cu	SACs	HAADF-STEM, EDS, PL, UV-vis, XPS, FTIR, SPV, fs-TAS, EPR, ICP, DFT	Photocatalytic hydrogen production	[96]
TiO_2_	Cu	SNCs	HAADF-STEM, EDS, XPS, ICP, UV-vis, PL, TRPL	Photocatalytic hydrogen production	[97]
TiO_2_	Pd	SACs	STEM, XPS, EPR	-	[71]
ZrO_2_	Ni	SACs, SNCs	AC HAADF-STEM, XANES, EXAFS, XPS, DFT	Photocatalytic CO_2_ Reduction to CO	[98]
ZrO_2_	Pt	SACs	AC-TEM, EDS, ICP, XANES, EXAFS, XPS, Raman	Photocatalytic CO_2_ Reduction to CO	[99]

Among metal oxides, zirconia ZrO_2_, a high conduction band semiconductor, is a promising candidate for photocatalytic procedures such as hydrogen production and CO_2_ reduction [132,133]. X. Xiong et al. [98] utilized a sol-gel approach to produce defect-rich zirconia with isolated Ni single atoms. The Ni loading ranges from 2 to 8 wt.%, depending on the weight ratio of Ni salts (Ni-SA-x/ZrO_2_). The researchers utilized HAADF-STEM microscopy to identify the aggregation state of Ni. Singe Ni atoms were present with dark spots in Z-contrast images for the Ni-SA-5/ZrO_2_ sample [98]. Additionally, the materials with different Ni loadings were evaluated using K-edge XANES and EXAFS. The Ni-Ni interactions of the first-shell Ni-Ni feature (2.1 Å) of metallic Ni foil and the second-shell Ni-Ni feature in NiO (2.5 Å), ref. [98] were absent in the Ni-SA-x/ZrO_2_ (x = 2, 5) photocatalysts, which indicates that Ni atoms were highly dispersed. On the contrary, in the sample with high Ni loading, the presence of the presence of Ni-Ni features in the R-space plots was observed [98], suggesting that Ni species aggregated into possible nanoclusters [98]. The photocatalysts, decorated with different Ni loadings, were evaluated in photocatalytic CO_2_ reduction to CO. The optimal photocatalyst for the highest CO yield was Ni-SA-5/ZrO_2_, which, according to the researchers, contained a large amount of single nickel atoms [98]. To support the experimental results, theoretical studies were performed, demonstrating that the atomically dispersed Ni sites reduce the energy barrier for CO_2_ to CO conversion through an adsorbed COOH intermediate while also inhibiting H_2_ desorption in the competing water-splitting reaction. As a result, the Ni single atom sites effectively promote both CO_2_ conversion and CO selectivity [98].

Another work employing zirconia substrate was reported by S. Dong et al. [99] in which single Pt atoms were anchored on amorphous ZrO_2_ nanowires. The amorphous support was prepared using a wet chemistry approach, followed by the incorporation of Pt SAs through an impregnated reduction process. Spherical aberration-corrected TEM was used to examine the aggregation state of the co-catalyst. Individual bright spots appeared on the image that were measured to be less than 1nm and assigned to the Pt single atoms. The EXAFS spectrum displayed a strong peak around 1.6 Å for Pt-SAs/ZrO_2_ [99], indicating Pt-O coordination, with no notable Pt-Pt bond signal between 2.5 and 3.0 Å [99], indicating that the Pt species on the ZrO_2_ were atomically dispersed. Photocatalytic CO_2_ reduction experiments were conducted, where Pt-SAs/ZrO_2_ exhibited the highest photocatalytic efficiency compared to crystal-ZrO_2_ and amorphous-ZrO_2_ nanowires. Overall, the researchers concluded that the amorphous ZrO_2_ nanowires contain numerous oxygen defects that help coordinate and stabilize the Pt single atoms [99]. These defects also promote the formation of Pt-O-Zr charge bridges, which improve the catalyst’s light absorption and the efficiency of photogenerated electron transport. As a result, these features enhance the effective use of photoelectrons, boost the formation of intermediate products, and facilitate CO desorption [99].

## 5. Concluding Remarks—Future Perspectives

Single atom catalysis provides remarkable efficiency and precise selectivity toward various photocatalytic applications, which is attributed to the maximized atomic utilization, distinctive active sites, and strong metal–support interaction (SMSI) between the co-catalyst and the semiconductor. Nevertheless, these systems face several challenges that limit their photocatalytic performance.

The synthesis of single atom co-catalysts (SACCs) poses significant challenges, as it requires control of the desirable metal loading while avoiding agglomeration on the semiconductor surface, uniformly dispersing single atoms or sub-nanoclusters, and establishing a robust interface to promote strong metal–support interactions. Perovskites possess promising photocatalytic properties, and furthermore, they appear to be stable and efficient substates to anchor and stabilize co-catalytic single atoms/sub-nanoclusters and/or quantum-sized small particles. However, due to their special chemistry, their synthesis, and the deposition of the co-catalyst on their surface, they become more complex compared to metal oxide substrates. Subsequently, in many cases, there is little control of the desirable aggregation state of the co-catalyst, and mostly, it is achieved via trial and error using different metal loadings. Although many synthesis processes have been utilized to produce these delicate materials, ranging from wet to dry methods, it is essential to establish more synthetic routes capable of controlling the atomic dispersion of single atoms or sub-nanoclusters, especially on complex substrates such as perovskites.

Furthermore, adequate characterization of the exact configuration of the co-catalyst has been an issue as it requires state-of-the-art machinery as well as stupendous analytical skills. In some cases, it leads to misunderstandings concerning the aggregation state of the co-catalyst on the catalyst’s surface. The most reliable set of techniques that have been established so far begins with HAADF-STEM, which provides a clear outlook on how the single atoms became anchored on the support’s surface. Then, X-ray absorption techniques are in place. XPS can illustrate the chemical states and electronic environments of the atoms involved. EXAFS gives data on bond lengths, coordination numbers, and the identity of the elements surrounding the absorbing atom. This information comprises the local atomic arrangement. XANES can reveal alterations in oxidation states, which further reveal the electronic environment of the atoms. EPR analysis may also assist in identifying single atoms if they possess unpaired electrons. Their sharp signals will serve as signatures for isolated atoms. Finally, comparing DFT simulations with experimental catalytic performance can offer further evidence of the presence and efficacy of isolated single atoms relative to established benchmarks, although reaching a definitive conclusion through this route is often more challenging than it may appear. Despite these techniques possibly being the best methods for identifying single atoms at present, they do not come without drawbacks and limitations. As previously discussed, it is most probable that the results of the anchoring process follow a distribution that ranges from single atoms to clusters and nanoparticles. STEM experiments can only provide local information that might not even represent the rest of the material. In addition, the STEM data are reliable only if the elements have a good enough Z factor to create adequate contrast. The techniques of the XAS umbrella often struggle with background signals and overlapping peaks, resulting in unreliable data. Similar problems apply to the XPS, where overlapping peaks may result in misinterpretation of oxidation states. EPR spectroscopy can provide detailed data and even quantitative analysis on single atoms and sub-nanoclusters, given (however) that the elements have unpaired electrons in order to be EPR-visible. In order for the subdivision of single atom co-catalysts to expand and the field of catalysis to achieve new heights, these techniques must evolve and combine to provide sufficient and reliable data on the results of the anchoring process.

In current literature, single atoms are preferred to in the majority of catalytic applications; however, a comprehensive study concerning the function of SA/SNC/QSSPs in a specific photocatalytic procedure is lacking. Different configurations of the co-catalyst lead to different electronic properties, from discrete energy multiplicities in small nanoclusters to semi-discrete bands in quantum-sized-small particles and full-periodic density of states in large nanoparticles. Thus, the optimal co-catalyst configuration should be selected and examined carefully to fit the requirements of each individual photocatalytic application.

## Figures and Tables

**Table 3:** Some advantages/limitations of main characterization techniques used to identify and distinguish single atoms and sub-nanoclusters.

Characterization Technique	Advantages	Limitations
AC HAADF-STEM	Visual mapping	Expensive equipment may damage the sample
XAS	Local structural and electronic information	Requires synchrotron facilities and advanced modeling software
XPS	Surface sensitive	Sample-destructive, unreliable for low-loading
EPR	High resolution	Limited to paramagnetic species
CO-DRIFT	Can distinguish single atoms from small nanoclusters	Signals often overlap, may react with the sample

## Data Availability

Not applicable.
